# Thyroid nodule and lymph node metastasis assessment from ultrasound images using deep learning

**DOI:** 10.3389/fnins.2025.1684104

**Published:** 2025-11-03

**Authors:** Xiaohui Zhao, Gang Zhang, Xueqin Shen, Diansheng Jin, Yanrong Wei, Yu Zhang, Xin Liu, Yang Liu, Dongfang Yang, Huiying Xiao, Xianquan Shi, Xiaoguang Yang

**Affiliations:** ^1^Department of Radiology, Affiliated of Hohhot First Hospital, 5th Clinical College of Inner Mongolia Medical University, Hohhot, China; ^2^Beijing Institute of Technology, Beijing, China; ^3^The Geriatric Clinic of the Second Medical Center of the People's Liberation Army General Hospital, Beijing, China; ^4^School of Information and Communication, Guilin University of Electronic Technology, Guilin, China; ^5^Department of Obstetrics and Gynecology, Affiliated of Hohhot First Hospital, Hohhot, Inner Mongolia, China; ^6^Department of Ultrasound, Beijing Friendship Hospital, Capital Medical University, Beijing, China

**Keywords:** thyroid nodules, metastatic malignant thyroid nodules, computer-assisted diagnosis, ultrasound imaging, medical image analyzing

## Abstract

**Objectives:**

The preoperative differentiation of thyroid nodules into benign thyroid nodules (BTN), non-metastatic malignant thyroid nodules (NMTN), and metastatic malignant thyroid nodules (MMTN) is critical for guiding clinical management strategies. Ultrasound (US) examinations frequently exhibit diagnostic inconsistencies due to operator-dependent variability. Computer-assisted diagnosis (CAD), an artificial intelligence (AI) model based on convolutional neural networks (CNNs), can help overcome inconsistencies in US examination outcomes by leveraging large-scale ultrasound imaging datasets to improve classification accuracy. Our study aimed to establish and validate this AI-powered ultrasound diagnostic model for precise preoperative discrimination among BTN, NMTN, and MMTN.

**Methods:**

A total of 209 patients (BTN = 66, NMTN = 15, and MMTN = 128) were consecutively identified and enrolled from a multi-center database. A subset of 195 patients (BTN = 60, NMTN = 15, and MMTN = 120) was selected for final analysis. These patients were divided into two groups: a training set (BTN = 50, NMTN = 11, and MMTN = 100) and a testing set (BTN = 10, NMTN = 4, and MMTN = 20). A total of 3,537 ultrasound images from the 195 patients were preprocessed by normalizing grayscale values and reducing noise. The processed images were then input into the AI model, which was trained to classify thyroid nodules. The model’s performance was evaluated using the testing set and assessed through receiver operating characteristic (ROC) curve analysis and the confusion matrix. Finally, the diagnostic accuracy of the AI model was compared with that of radiologists to determine its clinical utility in ultrasound-based diagnosis.

**Results:**

Compared to junior and senior radiologists, the AI model achieved near-perfect AUC values of 0.97 (BTN), 0.99 (NMTN), and 0.96 (MMTN), significantly outperforming the senior radiologist’s AUCs (0.88 for NMTN) and the junior radiologist’s weaker discrimination. In addition, the accuracy of this model was higher than all ultrasound radiologists (95% vs. 73 and 84% for the junior radiologist and senior radiologist, respectively).

**Conclusion:**

The AI-based ultrasound imaging diagnostic model showed excellent performance in differentiating BTN, NMTN, and MMTN, supporting its value as a diagnostic tool for the clinical decision-making process.

## 1 Introduction

The thyroid gland, a small endocrine organ in the anterior neck, produces triiodothyronine (T3) and thyroxine (T4), which are essential for regulating systemic metabolism and somatic growth (Khachnaoui et al., 2018). Abnormal cellular proliferation within the gland can lead to the formation of thyroid nodules, which are radiologically distinguishable and broadly categorized as benign thyroid nodules (BTN) and malignant thyroid nodules (MTN; Cooper, 2009; Acharya et al., 2014). Thyroid nodules are highly prevalent, affecting up to 67% of adults worldwide, with the majority requiring no invasive treatment. However, malignant transformation, though less frequent (5–15%), can lead to aggressive behaviors—such as in papillary thyroid carcinoma, where lymphatic spread occurs in 20–50% of cases. Lymph node metastasis (LNM) significantly increases the risk of locoregional recurrence, distant metastasis, and disease-related mortality (Zhuang et al., 2018; Ren et al., 2019), highlighting the need for accurate and early differentiation between BTN and MTN.

Fine-needle aspiration biopsy (FNAB) is the non-surgical gold standard for detecting malignancy (Acharya et al., 2014; Lamartina et al., 2016). However, this invasive diagnostic modality carries inherent limitations, including procedural costs, patient discomfort, and psychological distress, while simultaneously increasing healthcare system burdens. Cross-sectional imaging techniques such as computed tomography (CT) and magnetic resonance imaging (MRI) are increasingly used for preoperative staging based on the tumor, node, and metastasis (TNM) classification (Ma et al., 2019). However, CT offers limited soft-tissue resolution, while MRI—though superior in soft-tissue contrast and neural assessment—is hindered by high cost, motion artifacts, and radiation concerns (Son et al., 2018; Lee et al., 2008; Maraghelli et al., 2021). In contrast, conventional ultrasound (US) is widely adopted as the first-line imaging tool due to its real-time capability, accessibility, and cost-effectiveness (Acharya et al., 2014; Wettasinghe et al., 2019).

Unfortunately, US diagnosis remains highly operator-dependent, with inconsistent feature interpretation and a lack of standardization leading to substantial interobserver variability (Hoang et al., 2018). To mitigate these issues, computer-assisted diagnosis (CAD) systems, particularly those based on convolutional neural networks (CNNs), have emerged as valuable tools for offering objective second opinions (Prochazka et al., 2019; Nugroho et al., 2021; Guan et al., 2019; LeCun et al., 2015; Yao et al., 2025). CNNs show remarkable capability to perform more particularized analysis and integrate massive amounts of data at high speeds but low cost without explicit feature definition (Yao et al., 2020; Fukushima and Miyake, 1982; Bakator and Radosav, 2018; Suzuki, 2017). Recent years have seen increased efforts to develop CNN-based CAD systems for distinguishing BTN from MTN (Sai Sundar et al., 2019; Liu et al., 2017a,b; Sun et al., 2019; Mei et al., 2018), showing potential to enhance diagnostic consistency while remaining cost-effective. Despite these advances, few studies have focused on predicting lymph node metastasis in malignant thyroid nodules by CAD systems—a crucial prognostic factor that strongly influences treatment planning and patient survival (Zhang et al., 2021; Wang J. et al., 2023; Wang Z. et al., 2023; Kuo et al., 2022; Yao et al., 2022). This gap underscores the need for further research into AI-assisted evaluation of metastatic potential in thyroid cancer.

Drawing upon these insights, we integrate conventional AI-driven methodologies with thyroid US images to develop an AI diagnosis architecture for addressing this gap. This architecture aims to accurately classify thyroid nodules into three categories: benign (BTN), non-metastatic malignant (NMTN), and metastatic malignant (MMTN). The performance of this architecture is rigorously validated across large-scale, multi-center, prospective cohorts and compared with experienced radiologists. Additionally, we systematically evaluate the synergistic potential of this AI system as a decision-support tool to augment diagnostic accuracy and consistency in clinical practice. This study pioneers a metastasis-stratifying AI system for thyroid nodule diagnosis, enhancing diagnostic consistency and therapeutic decision-making through robust AI-radiologist collaboration, thereby advancing precision oncology in thyroid cancer management.

## 2 Materials and methods

**Figure 1:**
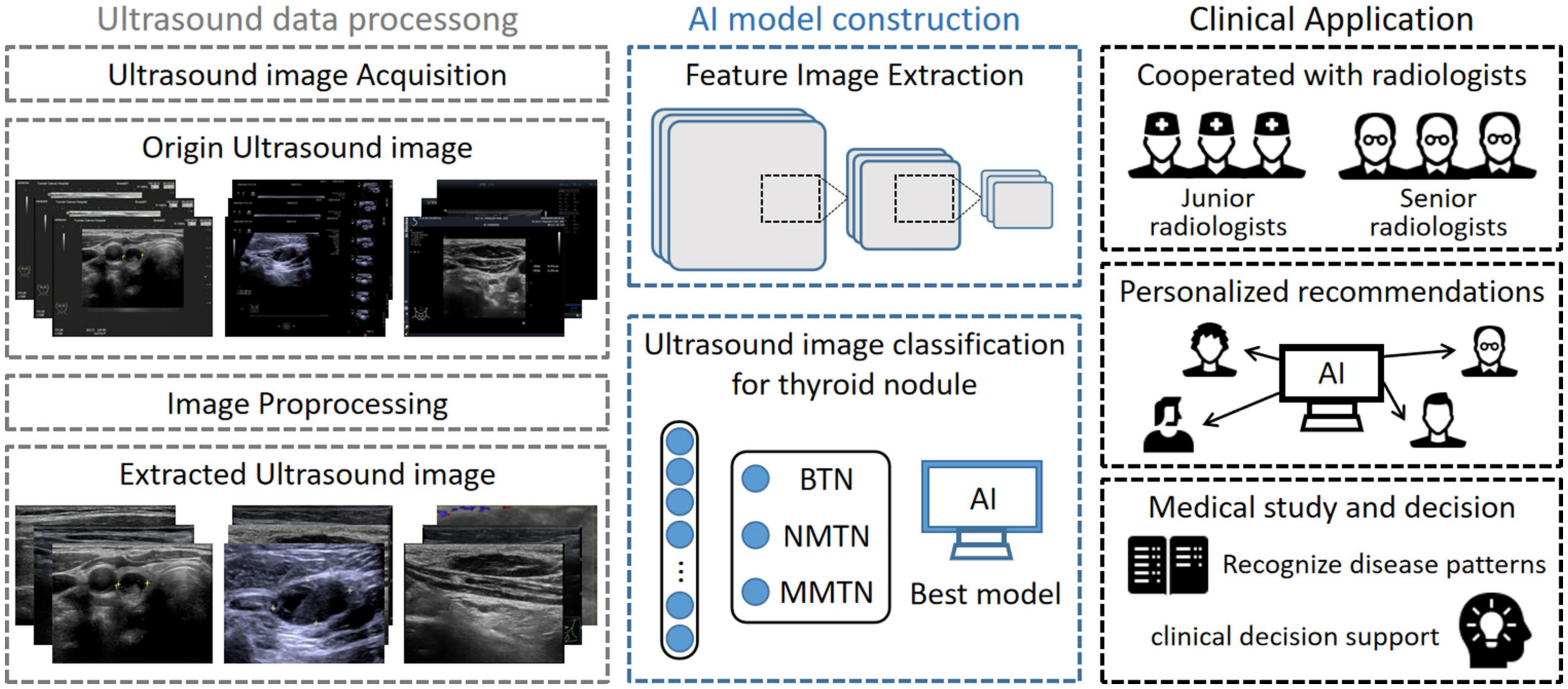
Schematic workflow of our study.

In this section, we describe the key methodological components of this investigation, including patient recruitment criteria, ultrasound imaging acquisition and processing, AI model construction, and statistical evaluation metrics. The schematic workflow of this multi-center study is presented in Figure 1.

This study was conducted in accordance with the Checklist for Artificial Intelligence in Medical Imaging (CLAIM) checklist, a reporting guideline for AI in medical imaging. The completed checklist is provided as Supplementary material.

### 2.1 Patients

This retrospective study was approved by the Hohhot First Hospital and The Third Affiliated Hospital of Kunming Medical University. The requirement for the patients’ informed consent was waived. A total of 209 patients (BTN = 66, NMTN = 15, and MMTN = 128) were consecutively identified by referring to the pathology database from our institution during the period from January 2015 to March 2023. The inclusion criteria of patients were as follows: (1) either BTN, NMTN, or MMTN was pathologically confirmed, (2) patients who underwent preoperative US examination, and (3) images were clear without motion or artifacts and were conducive to analysis. The exclusion criteria for patients were as follows: (1) those who had received treatment related to the lesion before the US examination, including surgery, transcatheter arterial chemoembolization (TACE), radiofrequency ablation, chemotherapy, radiotherapy, or targeted drug therapy; (2) those with inflammatory lesions; (3) patients with missing important medical records or laboratory results of individuals with malignancies; and (4) unqualified image quality, such as annotated information or incomplete lesion area. Finally, 195 patients (BTN = 60, NMTN = 15, and MMTN = 120) were picked from the pathology database. These were randomly divided into a training set (BTN = 50, NMTN = 11, and MMTN = 100) and a testing set (BTN = 10, NMTN = 4, and MMTN = 20).

### 2.2 Ultrasound image acquisition and filtering

Ultrasound examinations were conducted between 2015 and 2023 by certified radiologists with over 5 years of experience in thyroid ultrasonography, using four commercially available systems: DC-8 (Mindray), Logic E9 (GE), HD15 (Philips), and IU22 (Philips). All devices were equipped with high-frequency linear array probes (6–14 MHz) to ensure basic imaging consistency, though technological advancements during this study period likely introduced variations in hardware performance and imaging algorithms across newer and older equipment models.

To mitigate potential confounding factors from device heterogeneity, standardized imaging protocols were implemented throughout the study period. These included uniform probe pressure application, optimal coupling agent usage, and consistent adjustment of image gain and focus position according to established thyroid imaging guidelines. For this study, two-dimensional ultrasound images were selected and cropped.

### 2.3 Ultrasound image preprocessing

We obtained two-dimensional ultrasound images of the thyroid nodules from the Picture Archiving and Communication Systems (PACS) and removed all patient-identifying information from the ultrasound images. Before utilizing these ultrasound images, we performed preprocessing to enhance their usability and efficiency. The data preprocessing workflow consisted of three main steps. First, contrast and brightness enhancement is performed to improve image visibility, ensuring that subtle lesion regions receive greater attention from the model. Second, normalization standardizes image data by scaling pixel values to a consistent range, which facilitates faster model convergence. Finally, data augmentation is used to address the significant class imbalance observed in the dataset, which could cause the AI model to favor classes with more samples. Techniques such as rotation, flipping, and adjustments in brightness and contrast are used to increase sample diversity and improve the model’s generalization ability.

The same preprocessing was applied to the testing set. Two-dimensional ultrasound images of thyroid nodules were embedded into the AI model for training and analysis, and the model’s prediction results were recorded. To optimize memory usage and training time, the image resolution was cropped to 224 × 224. This resolution, pretrained on ImageNet, aligns with the default input resolution of the network model.

### 2.4 Methodology of AI diagnostic architecture

**Figure 2:**
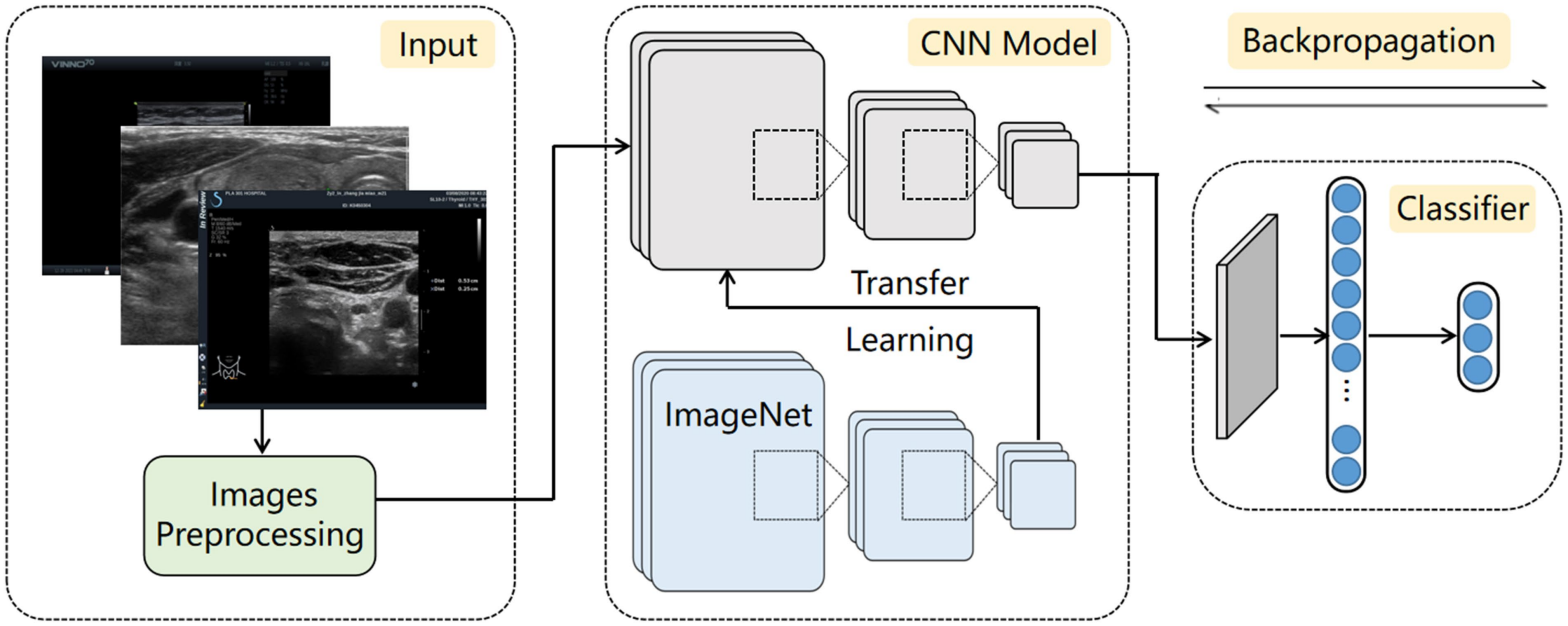
Workflow of the AI diagnosis architecture. Ultrasound images from participating institutions are divided into two groups (training set and testing set). Ultrasound images are processed by normalizing to grayscale and reducing noise. The processed images are then imported into the CNN model for training. Then, the images are predicted from the testing set, and the performance of the CNN model is evaluated.

The detection process of thyroid ultrasound images, as illustrated in Figure 2, consists of three main components: an input module, a convolutional neural network (CNN) model, and a classifier. Preprocessed thyroid ultrasound images are first input into the CNN, which acts as a feature extractor to capture discriminative features from various types of images. To enhance the model’s adaptability to small-scale datasets, transfer learning was used by initializing the CNN with weights pretrained on large public datasets. The pretrained model was fine-tuned using a low learning rate strategy, with specific layers unfrozen to allow feature specialization for thyroid ultrasound characteristics. Finally, a multilayer perceptron (MLP) adopted from the transformer architecture serves as the classifier. By modeling global dependencies within the feature maps, the MLP compensates for the local receptive field limitation of CNNs, thereby improving overall classification performance.

#### 2.4.1 VGG-19

**Figure 3:**
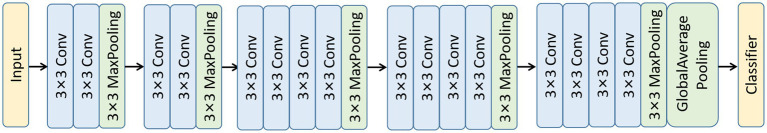
Structure of VGG-19. A VGG-19 is composed of a convolutional layer and a pooling layer.

For the selection of the CNNs, we utilized VGG-19 (Wen et al., 2019) as the deep learning model to extract image features. The primary advantage of the VGG-19 network lies in its systematic and hierarchical architecture. By increasing depth and stacking numerous 3 × 3 small convolutional kernels, it gradually extracts detailed features from images, enhancing the model’s feature representation capability and recognition accuracy. In essence, replacing large-scale convolutional kernels with stacked 3 × 3 kernels effectively increases the network’s depth and width. Furthermore, the VGG-19 structure is simple and easily transferable to other computer vision tasks. It achieves high accuracy while maintaining good generalization performance across different datasets (Figure 3).

#### 2.4.2 Classifier

**Figure 4:**
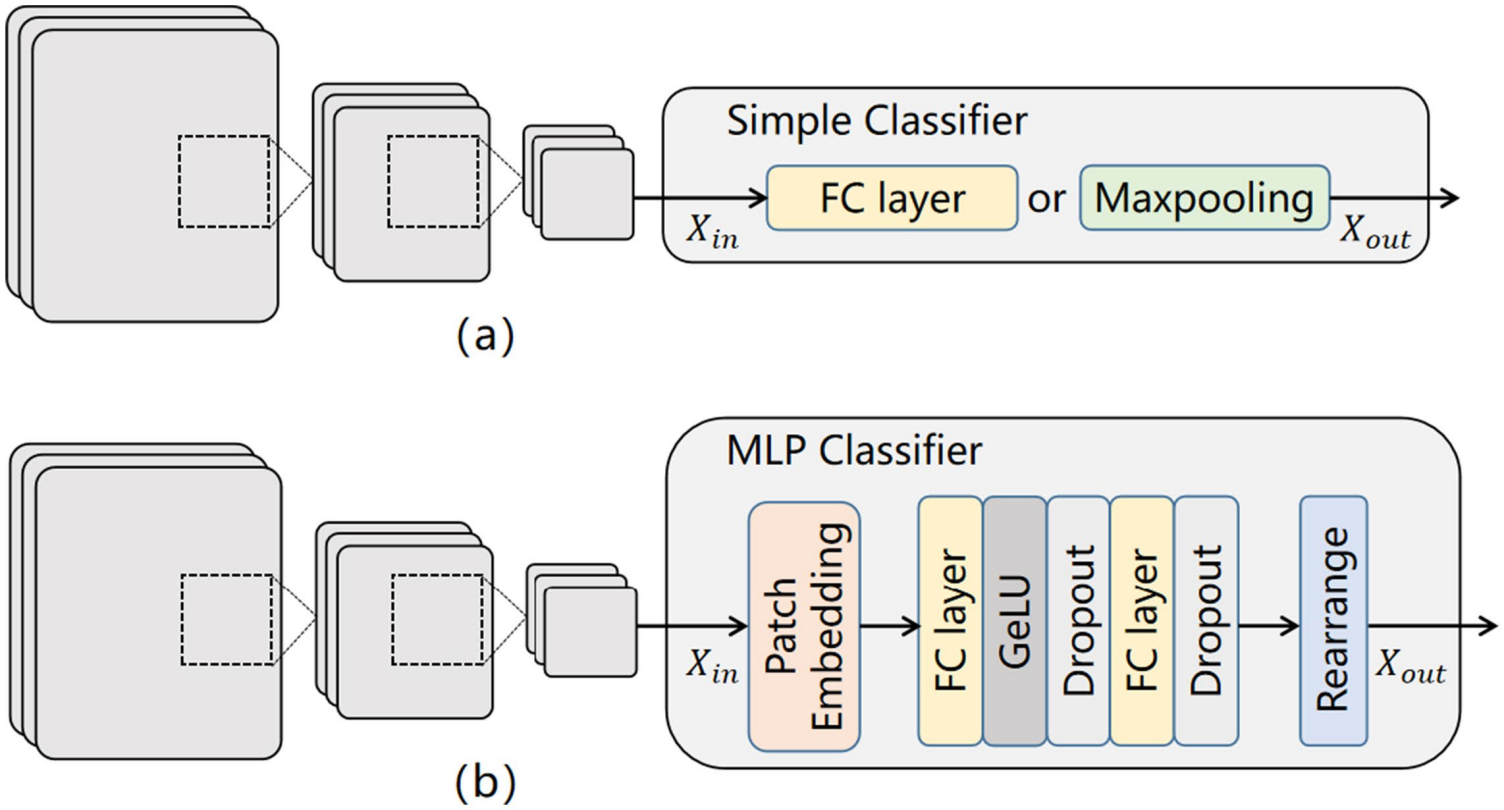
Comparison of classifier structures: **(a)** classifier structure of CNNs; **(b)** our classifier structure.

It is well known that the classifiers in CNNs are typically composed of fully connected (FC) layers or max-pooling functions (Figure 4A). These approaches utilize small convolutional kernels to capture local features of the ultrasound images, often overlooking the importance of global dependencies. Additionally, Tolstikhin (2021) found that the capability of modeling global dependencies can be achieved solely through the multilayer perceptron (MLP) in a transformer. As a result, we introduced an MLP classifier to address the above limitations and integrated it with the local feature extracted by CNNs to improve the model’s predictive performance for thyroid nodules. Figure 4B illustrates the structure of this module.

First, the feature images $X_{\text{in}}\in {\mathbb{R}}^{H\times W\times C}$ are entered into the Patch Embedding module, where they are divided into multiple small image patches and enriched with positional embeddings to facilitate the extraction of global features. Here, (H, W) represents the resolution of the original image, and C denotes the initial dimension. Subsequently, these image patches are fed into the multilayer perceptron (MLP) module, which comprises multiple fully connected (FC) layers, activation functions (GeLU), and dropout layers. GeLU and dropout layers are used to prevent overfitting and to improve training accuracy. The FC layer serves to transform the two-dimensional feature patches into one-dimensional vectors. Finally, during the rearrangement phase, these feature vectors are assembled into vectors of specific dimensions, enabling the classification of thyroid ultrasound images.

### 2.5 Statistical analysis

The receiver operator characteristic (ROC) curve was used to evaluate the AI model’s discrimination ability for parotid gland tumors. The ROC curve is a synthetic indicator that responds to variable sensitivity and mutable specificity. Furthermore, the F1-score was calculated to further assess the performance of the AI model. The relevant calculation formula is as follows:


$$\text{Precision}=\frac{\mathrm{TP}}{\mathrm{TP}+\mathrm{FP}}$$

$$\text{Sensitivity}/\text{Recall}=\frac{\mathrm{TP}}{\mathrm{TP}+\mathrm{FN}}$$

$$F1\;\text{Score}=\frac{2\cdot \text{precision}\cdot \text{recall}}{\text{precision}+\text{recall}}$$

$$\text{Accuracy}=\frac{\mathrm{TP}+\mathrm{TN}}{\mathrm{TP}+\mathrm{TN}+\mathrm{FP}+\mathrm{FN}}$$

$$\text{Jaccard Score}=\frac{\mathrm{TP}}{\mathrm{TP}+\mathrm{FP}+\mathrm{FN}}$$

$$\text{Kappa Score}=\frac{\text{Accuracy}-p_{e}}{1-p_{e}}$$

$$p_{e}=\frac{\left(\mathrm{TP}+\mathrm{FP})(\mathrm{TP}+\mathrm{FN})+(\mathrm{FN}+\mathrm{TN})(\mathrm{FP}+\mathrm{TN}\right)}{{\left(\mathrm{TP}+\mathrm{TN}+\mathrm{FP}+\mathrm{FN}\right)}^{2}},$$

where TP is true positive, indicating that the image is correctly classified by the classification algorithm; FN is false negative, indicating that the image is wrongly classified by the classification algorithm into other categories; TN is true negative, indicating that the classification algorithm correctly classifies non-category images into other categories; and FP is false positive, indicating that the classification algorithm incorrectly classifies non-category images into such categories.

### 2.6 Diagnostic methodology of radiologists

Two radiologists were invited to participate in the diagnostic process, including a junior radiologist with 2 years of clinical experience in thyroid ultrasonography and a senior radiologist with over 5 years of specialized experience in the same field. To ensure the objectivity of their diagnoses, the pathological results of the thyroid nodules (serving as the gold standard for final diagnosis) were concealed from both radiologists.

In terms of diagnostic basis, the radiologists primarily relied on thyroid ultrasound images as the core reference for judgment—this finding aligns with standard clinical practice for preoperative thyroid nodule assessment. When necessary, they additionally incorporated limited routine clinical data (e.g., basic patient demographics and non-specific clinical symptoms), but such data were strictly limited to information that was not directly related to the gold standard (pathological results) and would not interfere with the independent judgment based on ultrasound findings. Finally, both radiologists followed the diagnostic criteria outlined in the American College of Radiology (ACR) Thyroid Ultrasound Imaging Reporting and Data System (TI-RADS) to classify the nodules into three categories: BTN, NMTN, and MMTN.

## 3 Results

### 3.1 Clinical characteristics

From January 2015 to March 2023, 209 FLLs were retrospectively collected and divided into the training set A (*n* = 161) and testing set (*n* = 34). A total of 4,016 ultrasound images were acquired from our institution for the training and testing sets; however, 479 images were excluded based on the inclusion and exclusion criteria. The final dataset comprised 3,537 ultrasound images from 195 patients. The dataset for the 34 patients was used as the testing set to evaluate our model. Baseline characteristics and clinicopathological information on tumor size and distribution are provided in Table 1.

**Table 1:** Comparison of diagnosis performance between radiologists and the AI model.

Method	Accuracy	F1-score	Kappa score	Jaccard index	Recall
Junior radiologist	0.7321	0.7287	0.5974	0.5796	0.7321
Senior radiologist	0.8369	0.8366	0.7540	0.7209	0.8369
AI architecture	0.9501	0.9511	0.9250	0.9075	0.9501

### 3.2 Comparison of the results between junior and senior radiologists

As shown in Table 1, the diagnostic results between junior and senior radiologists showed significant differences in all evaluated metrics. The senior radiologist presented superior performance, achieving an accuracy of 0.8369 compared to 0.7321 for the junior radiologist. This advantage is consistently reflected across other key indicators: the senior radiologist attained an F1-score of 0.8366 (vs. 0.7287), a kappa score of 0.7540 (vs. 0.5974), a Jaccard index of 0.7209 (vs. 0.5796), and a recall of 0.8369 (vs. 0.7321). The results clearly indicated that more experienced radiologists exhibit substantially better diagnostic consistency and reliability across all measured parameters.

**Figure 5:**
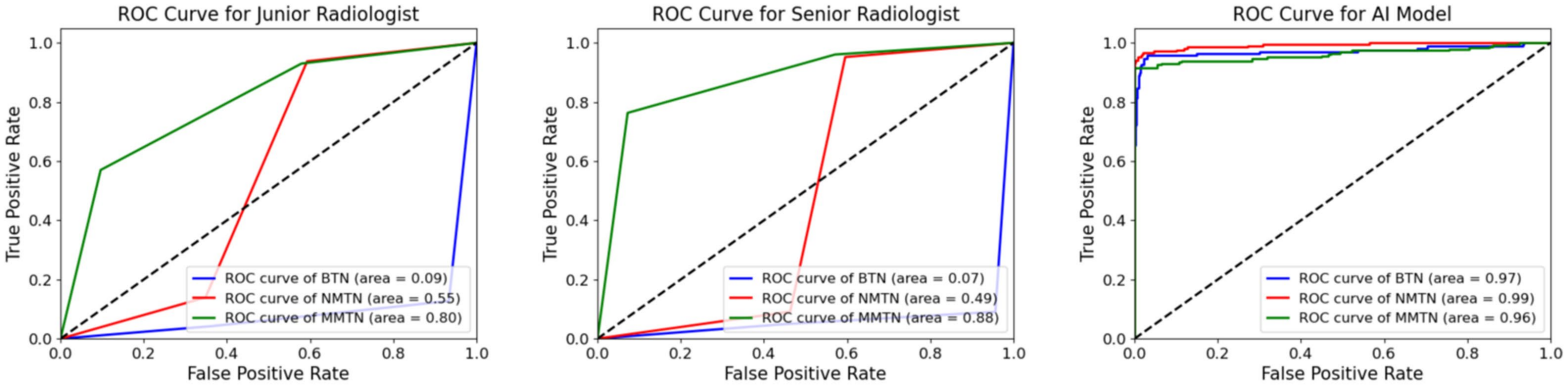
Comparison of AUC value between radiologists and the AI model. The ROC curves for the diagnostic performance of the junior radiologist in detecting BTN, NMTN, and MMTN (left). The ROC curves for the diagnostic performance of senior radiologists in detecting BTN, NMTN, and MMTN (middle). The ROC curves for the diagnostic performance of the AI model in detecting BTN, NMTN, and MMTN (right).

**Figure 6:**
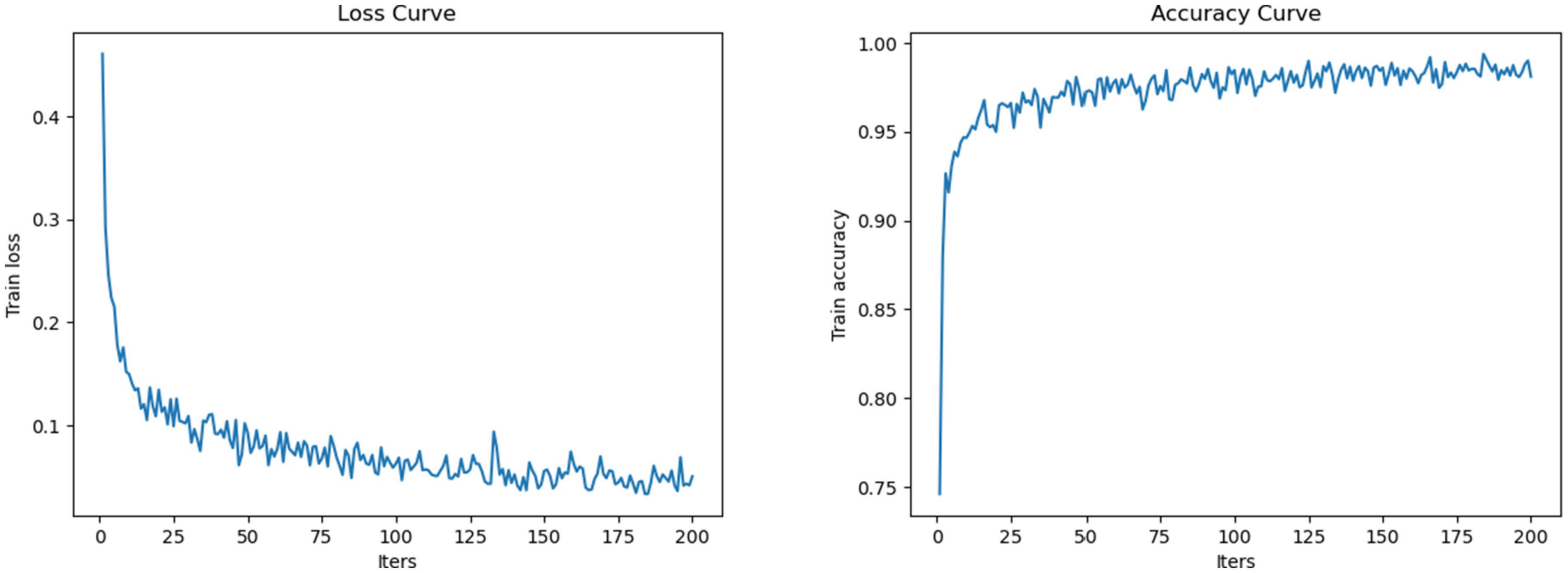
Loss and accuracy curve.

Figures 5, 6 show more characteristics across different nodule classifications. For the junior radiologist, the ROC curve showed AUC values of 0.09 for benign thyroid nodules (BTN), 0.55 for non-metastatic malignant thyroid nodules (NMTN), and 0.80 for metastatic malignant thyroid nodules (MMTN), indicating progressively better discrimination capability with increasing malignancy severity. The junior radiologist presented different diagnostic patterns, with confusion matrices showing correct identification for 144 of 165 (90%) BTN cases, 166 of 208 (79%) NMTN cases, and 130 of 228 (57%) MMTN cases. The comparative analysis revealed that the senior radiologist maintained more balanced performance across all three categories, while the junior radiologist showed particularly low discrimination for benign cases (AUC 0.09) but improved detection of metastatic cases (AUC 0.80).

### 3.3 Results comparison between AI model and radiologists

**Figure 7:**
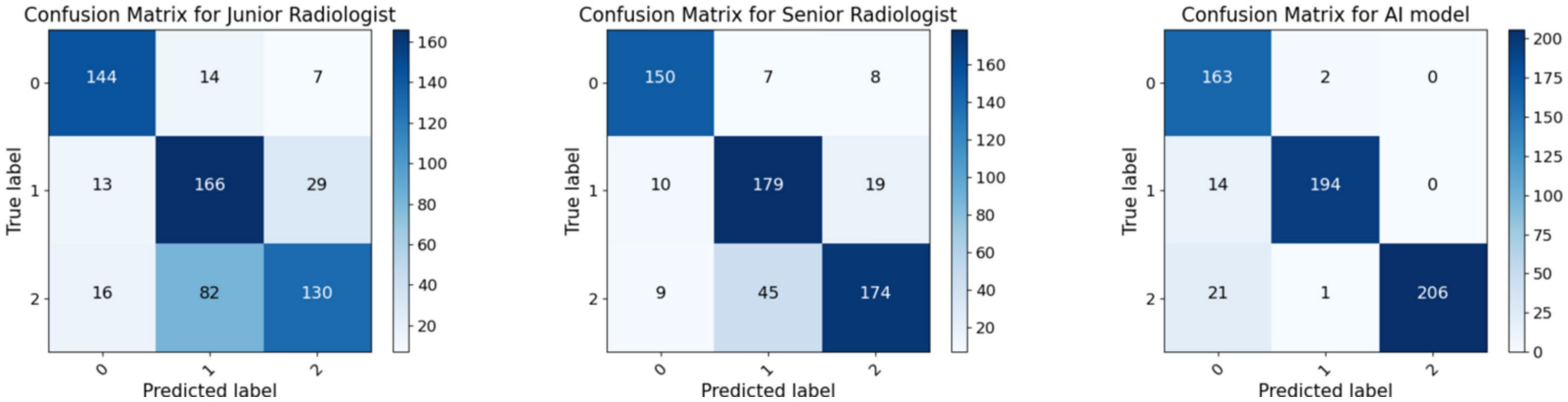
Comparison of the confusion matrix between radiologists and the AI model. The confusion matrix for the diagnostic performance of the junior radiologist in detecting BTN, NMTN, and MMTN (left). The confusion matrix for the diagnostic performance of senior radiologists in detecting BTN, NMTN, and MMTN (middle). The confusion matrix for the diagnostic performance of the AI model in detecting BTN, NMTN, and MMTN (right).

As shown in Table 1, the diagnostic performance of the AI model differed clearly among the three groups. The junior radiologist achieved an accuracy of 0.7321 and an F1-score of 0.7287, while the senior radiologist showed improved results with an accuracy of 0.8369 and an F1-score of 0.8366. The AI architecture outperformed both radiologists, attaining the highest scores across all metrics, including accuracy (0.9501), F1-score (0.9511), and kappa score (0.9250). The recall rates for all three groups matched their respective accuracy scores, indicating consistent diagnostic sensitivity. These results demonstrate a progressive enhancement in diagnostic precision from junior to senior radiologists, with the AI architecture achieving the highest performance. Moreover, the loss and accuracy curve (Figure 7) during training demonstrate stable convergence behavior without evidence of overfitting.

Figures 5, 6 exhibit the superior diagnostic performance of the AI model than both junior and senior radiologists across all evaluation metrics. The model achieves near-perfect AUC values of 0.97 (BTN), 0.99 (NMTN), and 0.96 (MMTN), significantly outperforming the senior radiologist’s AUCs (0.88 for NMTN) and the junior radiologist’s weaker discrimination. In classification accuracy, the AI model (95.0%) surpassed both the senior radiologist (83.7%) and junior radiologist (73.2%). The confusion matrix further confirmed the AI’s precision, with 98.8% correct BTN (163/165), 93.3% correct NMTN (194/208), and 90.7% correct MMTN (206/228) classifications—markedly higher than the senior radiologist’s 91% BTN accuracy (150/165) and 76% MMTN accuracy (174/228). These results highlighted the AI model’s consistent advantage over human evaluators, particularly in challenging metastatic cases where radiologists showed higher misclassification rates. The balanced sensitivity and specificity of this model suggested strong potential for clinical decision support in thyroid nodule diagnosis.

**Figure 8:**
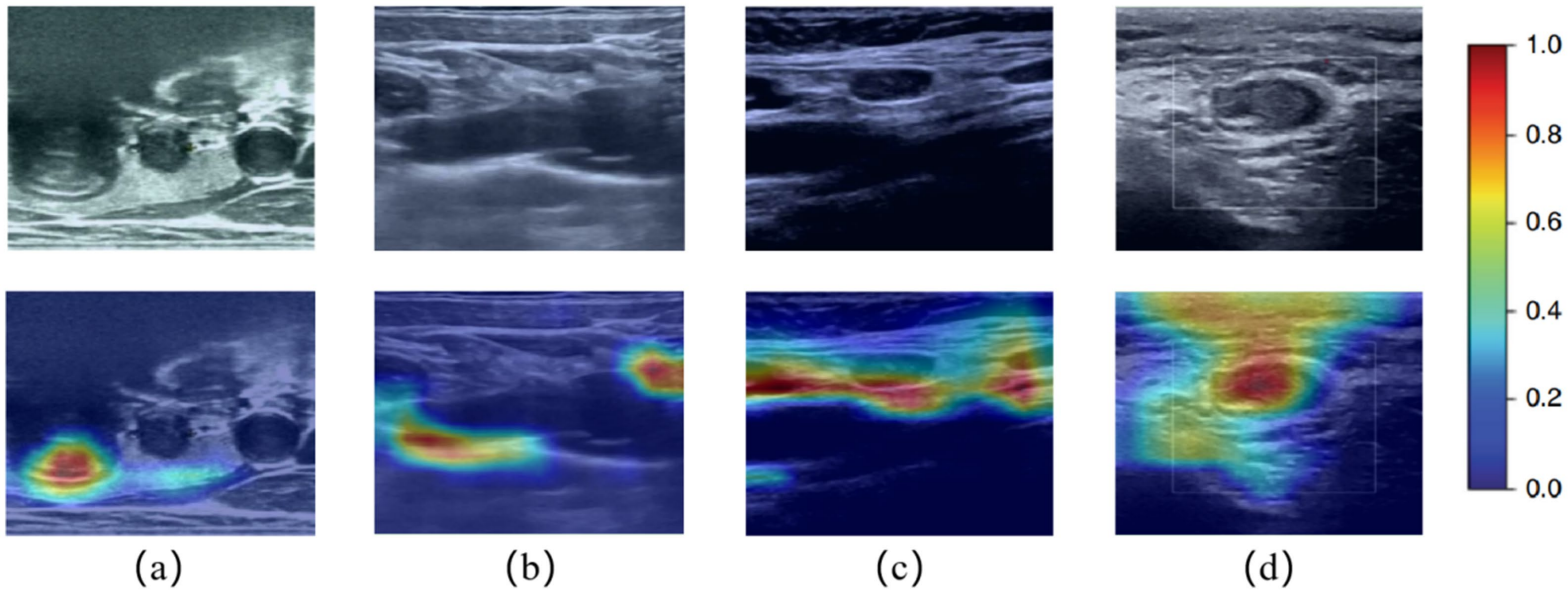
Ultrasound images and corresponding heatmaps of thyroid nodules. The heatmaps show the importance of the predictive image features of the AI architecture using different colors. Red and yellow represent the most powerful predictive areas of the tumor, and regions of blue and green show weaker predictive areas.

Surprisingly, in the grayscale US images, we found that the AI diagnostic architecture could predict the thyroid nodule from two locations: the border of the nodule and the low echo area in the nodule body (Figure 8). This capability significantly illustrates the effectiveness of the AI architecture. Heatmaps were generated using the weight file from the training process (Figure 8). The results in Figure 8 show that the regions concentrated with the highest predictive value are highlighted in red and yellow, whereas those with weaker predictive values are expressed as green and blue. This finding suggested that the AI architecture focuses on the most predictive image features of thyroid nodules.

### 3.4 Ablation study

In this section, we present the performance of the proposed classifier within our AI diagnostic architecture. Compared to a conventional classifier, we conducted an ablation study to explore the effectiveness of employing the MLP as the classifier for thyroid nodule diagnosis. Table 2 displays the test results of various metrics for two different classifiers on the dataset. The results demonstrate that the AI architecture equipped with the MLP classifier outperformed the conventional classifier across all evaluation metrics, indicating the effectiveness of our approach. This method shows promise in facilitating accurate differential diagnosis of BTN, NMTN, and MMTN.

**Table 2:** Comparison of diagnosis performance between different classifiers.

Classifier	Accuracy	F1-score	Kappa score	Jaccard index	Recall
Convention	0.9218	0.9295	0.8834	0.8679	0.9218
MLP	0.9501	0.9511	0.9250	0.9075	0.9501

## 4 Discussion

### 4.1 Experimental results

In this section, we first analyze the diagnostic results of the junior and senior radiologists, revealing significant disparities across all assessed metrics, as shown in Table 1. The senior radiologist demonstrated superior performance with an accuracy of 0.8369, compared to 0.7321 for the junior radiologist, representing a 14.3% relative improvement. This performance gap was consistently observed in other key indicators: the F1-score (0.8366 vs. 0.7287), Cohen’s kappa score (0.7540 vs. 0.5974), and the Jaccard similarity index (0.7209 vs. 0.5796), all showing approximately 15–20% enhancement for the senior practitioner. Notably, the recall rates perfectly matched the accuracy scores for both groups (0.8369 and 0.7321, respectively), suggesting consistent diagnostic sensitivity throughout the evaluation. These findings highlight the substantial impact of clinical experience on radiological diagnostic precision, particularly in complex case interpretations where senior radiologists’ expertise yielded significantly more reliable results.

The ROC curve (Figure 5) and confusion matrix (Figure 6) show distinct performance patterns between junior and senior radiologists in predicting benign thyroid nodules (BTN), non-metastatic malignant nodules (NMTN), and metastatic malignant thyroid nodules (MMTN). The junior radiologist demonstrated limited diagnostic capability for BTN cases (AUC = 0.09) while showing moderate performance in MTN classification (AUC = 0.55–0.80). Confusion matrices revealed significant misclassification patterns: the junior radiologist correctly identified 144 of 165 BTN cases (87% accuracy) but showed reduced accuracy in metastatic cases (130 of 228 MMTN correctly classified, 57% accuracy). For NMTN, the senior radiologist correctly diagnosed 179 of 208 cases (86% accuracy), representing a marked improvement over the junior counterpart. The ROC analysis demonstrated particularly strong discrimination capability for the senior radiologist in NMTN classification (AUC = 0.88), suggesting more reliable differentiation between malignant subtypes. However, both groups faced challenges in distinguishing metastatic cases, as evidenced by the 29 false negatives in MMTN classification by the senior radiologist, indicating persistent diagnostic difficulties with advanced disease states.

Subsequently, we compare this performance with our AI model. Table 1 shows a hierarchical pattern across all assessment metrics, with the CNN-based AI model demonstrating superior performance compared to both junior and senior radiologists. The AI model achieves exceptional classification accuracy (0.9501) and recall (0.9501), outperforming the senior radiologist by 13.5% and junior radiologists by 29.8% in the overall diagnostic precision. This performance advantage is consistently maintained across all evaluation metrics: F1-score (DL: 0.9511 vs. senior: 0.8366), Cohen’s kappa score (DL: 0.9250 vs. senior: 0.7540), and the Jaccard index (DL: 0.9075 vs. senior: 0.7209), indicating substantially better agreement with ground truth classifications. While the senior radiologist showed expected improvements over their junior counterpart (10.5% higher accuracy, 14.8% better F1-score), both human evaluators were significantly surpassed by the AI system. Additionally, the perfect alignment between accuracy and recall scores across all three evaluators suggested consistent diagnostic sensitivity in case identification. These results exhibited the potential of the AI model to augment clinical decision-making in thyroid nodule classification, particularly in challenging diagnostic scenarios where human performance typically declines.

Figures 5, 6 exhibit that the AI model achieved outstanding classification performance across all thyroid nodule subtypes, as evidenced by both ROC analysis and confusion matrix evaluation. This model showed near-perfect discriminative ability with AUC values of 0.97 for BTN, 0.99 for NMTN, and 0.96 for MMTN, indicating robust diagnostic capability regardless of lesion type. The confusion matrix revealed particularly impressive performance in NMTN identification, correctly classifying 194 of 208 cases (93.3% accuracy) while maintaining excellent performance for BTN (163 of 165 correct, 98.8% accuracy) and MMTN (206 of 228 correct, 90.7% accuracy). Specifically, our method demonstrated minimal false positives in BTN classification (2 cases) and showed balanced sensitivity across all categories, with only 21 false negatives observed in the challenging MMTN group. These results not only surpassed previously reported radiologist performance but also suggested the model’s particular strength in detecting early-stage malignancies (NMTN), where clinical diagnosis is often most challenging.

Moreover, the learning curves demonstrate stable convergence behavior without evidence of overfitting, as evidenced by the parallel trajectories of training and validation metrics. Throughout the 200-epoch training process, the model maintained close agreement between training and validation accuracy, while both loss curves showed synchronous reduction to minimal plateau values. This convergence pattern indicates the model achieved balanced learning with effective generalization to unseen data, as the final testing metrics closely matched the training set performance. The absence of diverging curves after epoch 150 particularly confirms the model’s robustness against overfitting.

### 4.2 Clinical implementation considerations and practical challenges

Beyond the technical performance metrics, several critical aspects related to clinical deployment must be addressed for successful real-world implementation. The integration workflow with existing Picture Archiving and Communication Systems (PACS) represents a fundamental consideration. Our proposed architecture would require seamless interoperability through standardized interfaces to minimize the disruption to clinical workflows. This finding entails developing middleware capable of efficiently processing imaging studies directly from radiologists’ workstations while maintaining data security and patient privacy.

Regarding computational requirements, the model’s inference time must meet clinical demands for real-time application. Our current implementation processes standard images within 1–2 s on GPU-accelerated hardware, which appears feasible for integration into typical radiology department workflows. However, institutions with high-volume caseloads may need to consider server-based deployment strategies with load balancing to maintain performance during peak hours.

The sustainability of AI architecture in clinical practice necessitates continuous model updating protocols. We recognize that diagnostic criteria and imaging technology evolve over time, potentially leading to model degradation. Therefore, this structured framework for periodic retraining using new data from participating institutions is coupled with rigorous validation against current clinical standards.

### 4.3 Handling diagnostic uncertainty and clinical decision support

A particularly important aspect for clinical adoption is the model’s behavior in ambiguous cases. Our architecture can directly output diagnostic probabilities, providing a confidence score to quantify uncertainty estimation. These scores are derived from model calibration analysis, enabling clinicians to gage the reliability of predictions. For cases falling below a predetermined threshold (e.g., probability scores between 0.4 and 0.6), the system flags them as requiring special attention and can optionally suggest additional imaging views or clinical correlation.

This uncertainty quantification directly supports clinical decision-making by identifying cases where human oversight is most valuable. Rather than presenting binary outcomes, the system provides probabilistic assessments that complement radiologists’ judgment. This approach acknowledges the inherent limitations of AI in navigating diagnostic gray areas while enhancing efficiency in straightforward cases. Future iterations will incorporate more sophisticated uncertainty estimation techniques, including Bayesian neural networks and ensemble methods, to further improve reliability in borderline cases.

## 5 Conclusion

In this study, we proposed an AI diagnostic architecture that precisely discriminates among benign thyroid nodules (BTN), non-metastatic malignant thyroid nodules (NMTN), and metastatic malignant thyroid nodules (MMTN) preoperatively. The architecture integrates the VGG-19 model with a multilayer perceptron (MLP) and, despite inherent limitations, demonstrates robust discriminative performance across these three types of thyroid nodules. Compared to the board-certified radiologists, our AI diagnostic architecture achieved statistically significant improvements in diagnostic accuracy. These findings indicate that the model holds strong potential as a clinical decision-support tool for preoperative characterization of thyroid lesions. However, prospective validation across diverse ethnic populations and different ultrasound scanner platforms is essential prior to clinical application.

## Data Availability

The raw data supporting the conclusions of this article will be made available by the authors, without undue reservation.
